# Gene fusions and gene duplications: relevance to genomic annotation and functional analysis

**DOI:** 10.1186/1471-2164-6-33

**Published:** 2005-03-09

**Authors:** Margrethe H Serres, Monica Riley

**Affiliations:** 1 Bay Paul Center for Comparative Molecular Biology and Evolution, Marine Biological Laboratory, 7 MBL Street, Woods Hole, MA02543-1015, USA

## Abstract

**Background:**

*Escherichia coli *a model organism provides information for annotation of other genomes. Our analysis of its genome has shown that proteins encoded by fused genes need special attention. Such composite (multimodular) proteins consist of two or more components (modules) encoding distinct functions. Multimodular proteins have been found to complicate both annotation and generation of sequence similar groups. Previous work overstated the number of multimodular proteins in *E. coli*. This work corrects the identification of modules by including sequence information from proteins in 50 sequenced microbial genomes.

**Results:**

Multimodular *E. coli *K-12 proteins were identified from sequence similarities between their component modules and non-fused proteins in 50 genomes and from the literature. We found 109 multimodular proteins in *E. coli *containing either two or three modules. Most modules had standalone sequence relatives in other genomes. The separated modules together with all the single (un-fused) proteins constitute the sum of all unimodular proteins of *E. coli*. Pairwise sequence relationships among all *E. coli *unimodular proteins generated 490 sequence similar, paralogous groups. Groups ranged in size from 92 to 2 members and had varying degrees of relatedness among their members. Some *E. coli *enzyme groups were compared to homologs in other bacterial genomes.

**Conclusion:**

The deleterious effects of multimodular proteins on annotation and on the formation of groups of paralogs are emphasized. To improve annotation results, all multimodular proteins in an organism should be detected and when known each function should be connected with its location in the sequence of the protein. When transferring functions by sequence similarity, alignment locations must be noted, particularly when alignments cover only part of the sequences, in order to enable transfer of the correct function. Separating multimodular proteins into module units makes it possible to generate protein groups related by both sequence and function, avoiding mixing of unrelated sequences. Organisms differ in sizes of groups of sequence-related proteins. A sample comparison of orthologs to selected *E. coli *paralogous groups correlates with known physiological and taxonomic relationships between the organisms.

## Background

*Eschericia coli *remains a useful resource to the genomic community as it provides important knowledge which can be applied to the analysis of most microbial genomes. Its central role devolves from two facts; first, the accumulated results of seven decades of laboratory experimentation have identified the function(s) of over half of its gene products; second being a metabolic generalist, *E. coli*'s metabolic functions are widely shared among other organisms.

Common practices of annotation rely, more than one might realize, on the accuracy of the annotation of *E. coli*'s genes. While searches for sequence matches to unknown genes usually yield a large number of matches, chances are high that firm functional information comes only from experimental studies on *E. coli*. Because annotations of genes do not always indicate that the assignments are derived, and because derived annotations are used serially for further annotation without experimental confirmation, many genes carry original *E. coli *annotations. It is therefore important to the entire genome-analyzing community that the data on *E. coli *gene products be as accurate as possible. Since the original GenBank deposit of *E. coli *K-12 (U00096), new and updated annotations are available at NCBI (U00096.2) and at more specialized databases including, ASAP [1], coliBASE [2], CyberCell [3], EchoBASE [4], EcoCyc [5], GenProtEC [6], and RegulonDB [7]. An effort is under way to coordinate the current *E. coli *annotations [8].

Over recent years, our work on the *E. coli *genome has led us to an appreciation of the pernicious role that gene fusions often play as troublemakers in function assignments and in relating groups of sequence similar proteins [9]. The fusion of two independently functioning genes results in the formation of a composite (multimodular) protein encoding for two independent functions located at separate parts of the protein. This type of fusion is not equivalent to the joining of protein domains, i.e. domains encoding binding sites for a cofactor or a substrate, which is seen in multidomain proteins. An example being the enzyme glyceraldehyde-3-phosphate dehydrogenase which according to the domain databases Pfam [10] and Superfamiliy [11] contains two domains, an NAD binding site and a dehydrogenase catalytic site. In our studies the entire protein including both domains represents one independent functional unit with one activity. Multidomain proteins are more prevalent and most often encode one overall function for the gene product [12].

Annotation involving transfer of function from composite proteins to sequence similar matches requires that the alignment regions be evaluated in order to determine whether all activities or only one of them should be assigned to the matching sequence. Currently fused proteins are themselves not always annotated to reflect that they encode more than one function, and rarely is the location of the separate functions indicated. Different combinations of fused genes are seen in the sequenced genomes, adding potential sources for annotation errors. Errors in functional assignments including those caused by fused genes have been noted years ago [13] and that such proteins may contribute to propagation of annotation errors in databases [14]. The fused proteins also interfere with the generation of sequence related protein groups as they link proteins based on their coexistence in a fused protein and not purely based on sequence similarity. Components of fused genes are often not sequence related, so generating protein groups without taking gene fusions into account may result in "mixed" groups of proteins with different sequence relatedness, functions and evolutionary histories.

Previous work has been published where we identified fused *E. coli *proteins from partial alignments between proteins encoded in the *E. coli *genome [15]. This work resulted in the identification of 287 multimodular proteins. As our analysis continued and more genome sequences were incorporated in our studies we realized that most of these identified multimodular proteins actually contained multiple domains and had one overall function. We have therefore revised our method of detecting fused proteins. We are making use of sequence information from 50 genomes including *E. coli *to detect proteins which are fused in the *E. coli *genome and are present as individual components in one of the other genomes. We have also made use of published experimental data on *E. coli *gene fusions. As a result the number of fused proteins in *E. coli *has been reduced to 109. The number of groups of sequence related proteins was also reduced from 609 to 490 since some of the previously identified groups are made up of protein domains catalyzing only part of an overall reaction. This work represents a revision of the state of fused proteins in the *E. coli *genome their affect on genome analysis both within *E. coli *and across genomes.

## Results

### Multimodular vs. multifunctional proteins

To prevent confusion, we define multimodular proteins as those seeming to result from gene fusion in which two independent proteins are connected. Multimodular proteins encode separate functions in different parts of the molecule. These functions might be the same if two like elements have fused, or as we see more often in *E. coli*, they differ in sequence and activity. Distinctly different, multifunctional proteins are defined as those that carry out more than one reaction or activity in the same part of the protein. Examples of such multifunctional proteins are encoded by the genes *cob*U, *bir*A, *ubi*G, *fol*D, *cys*G, *tes*A, and *ndk *(for details see gene products at GenProtEC [16]).

A protein that illustrates both properties is the FadB protein of *E. coli *[17,18]. FadB is a multimodular protein with N-terminal and C-terminal modules. Its N-terminal module is multifunctional with three activities that are catalyzed at the same active site and cannot be spatially separated along the length of the protein. The three activities are 3-hydroxybutyryl-CoA epimerase, delta(3)-cis-delta(2)-trans-enoyl-CoA isomerase, and enoyl-CoA hydratase. The C-terminal module of FadB encodes a single function, 3-hydroxyacyl-CoA dehydrogenase. Adding the N-terminal and C-terminal modules, there are 4 activities for the FadB protein.

### Identifying multimodular proteins in *E. coli*

In earlier work, before the genomic sequence of *E. coli *was completed, we saw that sequence similarity among its proteins was widespread [9,19]. After the entire sequence was available, we identified 287 *E. coli *proteins as being multimodular and encoded by fused genes [15,20]. The modularity of the proteins was inferred from the alignments among *E. coli *proteins. However, we have since found that many of these so-called multimodular proteins were proteins containing more than one domain and not more than one protein. Such multidomain proteins may appear to encode two functions but in reality encode two or more conserved motifs (i.e. DNA-binding and effector-binding domains of LysR type transcriptional regulators). By including sequence information from other genomes besides *E. coli *we were able to better distinguish fusions of complete proteins versus the more common fusions of protein domains. Of the 287 proteins previously identified as multimodulars only 70 remained as fused proteins in this study with the remaining representing domain fusions.

**Figure 1:**
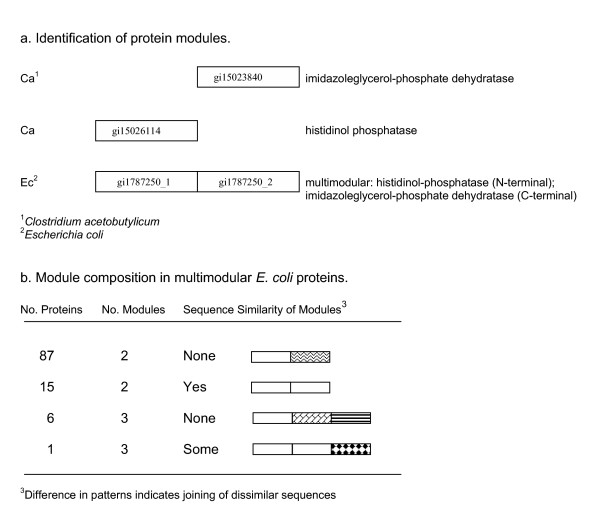
Identification and sequence similarity of multimodular *E. coli *proteins. (a) An *E. coli *protein (gi1787250) aligns with two smaller proteins from *C. acetobutylicum*, histidinol phosphatase (gi15026114) and imidazoleglycerol-phosphate dehydratatase (gi15023840). The *E. coli *protein represents a fused or multimodular protein encoding the two functions in separate parts of the protein as indicated by the two non-overlapping alignment regions. Based on the alignment regions, the *E. coli *protein is separated into two separate components, modules. The modules are identified with the extensions "_1" or "_2" to indicate their location in the gene product as N-terminal or C-terminal, respectively. (b) Sequence similarity between modules of the multimodular proteins is shown. No detectable similarity between the joined modules is indicated by a difference in the module patterns in the cartoon. Similarity is measured by Darwin and indicates that the proteins align at a distance of ≤ 200 PAM units over at least 83 amino acid residues or >45% of the length of the proteins. This level of similarity also reflects whether the modules belong to the same paralogous group.

In the present work, some of the fused proteins were identified by searching the literature for experimental data. Examples of *E. coli *proteins long known to contain multiple functions encoded at separate parts of the proteins include GlnE [21], MetL [22], ThrA [23], and TyrA [24]. We have collected such experimentally verified information over time [9], labeled as multimodular proteins with literature citations in our database GenProtEC [16]. Other multimodular proteins were identified by selected types of alignments between *E. coli *proteins and proteins encoded in 50 sequenced genomes. The component proteins of a multimodular protein may be unimodular and unfused in another genome. We looked for alignments between the larger potentially multimodular proteins in *E. coli *and smaller orthologous proteins that are homologous to only one of the modules (Figure 1a). Not all gene fusions of *E. coli *will be detected by this method. For instance elements of a fused gene may have diverged to the point where the component modules no longer have detectable similarity to their homologous counterparts, or the independently existing modules may have been lost from the gene pool of the 50 genomes analyzed, or the 50 organisms may contain only the multimodular form.

**Figure 2:**
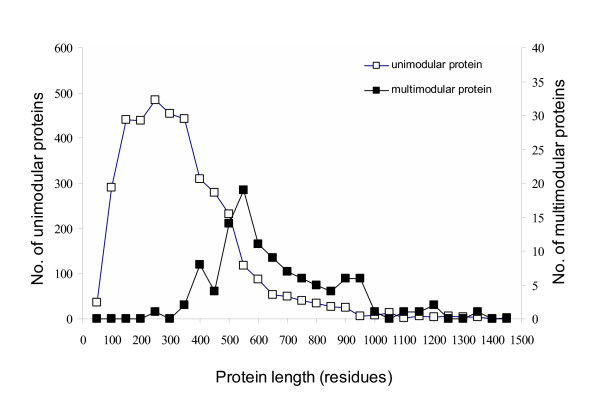
Size distribution for multimodular and single module proteins. The protein lengths in amino acid residues are shown for single module proteins (□) and for multimodular proteins (■). On average the multimodular proteins are longer than the unimodular proteins, 637 amino acids versus 314 amino acids. The length of a protein alone does not infer multimodularity and long single module proteins are seen.

In total we identified 109 *E. coli *proteins to be multimodular, with 101 containing two modules and 8 containing three modules. The largest number of multimodular proteins joined modules of dissimilar sequence (illustrated in Figure 1b). An abbreviated list of the modules and their functions is shown in Table 1. A complete list of the multimodular *E. coli *proteins is made available: ' [see Additional file 1]'. The remaining proteins, 97.5 % of the total, were considered to be unimodular. The average length of the multimodular proteins was 637 residues compared to 309 for the remaining proteins in the chromosome (Figure 2). Individual modules from the multimodular proteins were on average 300 residues long, similar to the length of the unimodular proteins. However, the size alone of a protein does not reflect multimodularity as we found many large proteins to be unimodular.

**Table 1:** Examples of multimodular *E. coli *proteins.

**Gene**	**Module**	**Start**	**End**	**Gty^1^**	**Module Function**
*thr*A	b0002_1	1	461	e	aspartokinase I, threonine sensitive
*thr*A	b0002_2	464	820	e	homoserine dehydrogenase I, threonine sensitive

*rib*D	b0414_1	1	143	e	diaminohydroxyphosphoribosylaminopyrimidine deaminase
*rib*D	b0414_2	147	366	e	5-amino-6-(5-phosphoribosylamino) uracil reductase

*put*A	b1014_1	1	569	e	bifunctional: transcriptional repressor (N-terminal); proline dehydrogenase, FAD-binding (C-terminal)
*put*A	b1014_2	618	1320	e	pyrroline-5-carboxylate dehydrogenase

*adh*E	b1241_1	1	400	e	acetaldehyde-CoA dehydrogenase
*adh*E	b1241_2	449	891	e	iron-dependent alcohol dehydrogenase

*thi*P	b0067_1	1	274	t	thiamin transport protein (ABC superfamily, membrane)
*thi*P	b0067_2	285	536	t	thiamin transport protein (ABC superfamily, membrane)

*mdl*A	b0448_1	1	310	pt	putative transport protein, multidrug resistance-like (ABC superfamily, membrane)
*mdl*A	b0448_2	314	590	pt	putative transport protein, multidrug resistance-like (ABC superfamily, ATP_bind)

*mod*F	b0760_1	1	260	t	molybdenum transport protein (ABC superfamily, ATP_bind)
*mod*F	b0760_2	261	490	t	molybdenum transport protein (ABC superfamily, ATP_bind)

*hrs*A	b0731_1	1	178	t	PTS family enzyme IIA, induction of ompC
*hrs*A	b0731_2	186	454	t	PTS family enzyme IIB, induction of ompC
*hrs*A	b0731_3	456	628	t	PTS family enzyme IIC, induction of ompC

*ato*C	b2220_1	1	125	r	response regulator
*ato*C	b2220_2	145	461	r	sigma54 interaction module of response regulator (EBP family)

evgS	b2370_1	1	935	e	histidine kinase of hybrid sensory kinase
evgS	b2370_2	953	1197	r	response regulator of hybrid sensory histidine kinase

*gln*G	b3868_1	1	120	r	response regulator, two-component regulator with GlnL, nitrogen regulation
*gln*G	b3868_2	139	469	r	sigma54 interaction module of response regulator (EBP family)

*kef*A	b0465_1	1	779	o	unknown function module of mechanosensitive channel
*kef*A	b0465_2	780	1120	t	mechanosensitive channel (MscS family)

*arg*A	b2818_1	1	293	o	acetylglutamate kinase homolog (inactive)
*arg*A	b2818_2	298	442	e	N-alpha-acetylglutamate synthase (amino acid acetyltransferase)

*ydc*R	b1439_1	1	117	pr	putative transcriptional regulator (GntR family)
*ydc*R	b1439_2	118	468	pe	putative amino transferase

*rnf*C	b1629_1	1	448	pc	Fe-S binding module of electron transport protein
*rnf*C	b1629_2	450	740	o	unknown function module of electron transport protein

^1^Gene product type: e, enzyme; pe, putative enzyme; r, regulatory protein; pr, putative regulatory protein; t, transport protein; pt, putative transport protein; pc, putative carrier protein; o, unknown function.

### Characteristics of multimodular proteins of *E. coli*

Table 2 shows some characteristics of the modules in the multimodular proteins. The majority of the *E. coli *modules, 90%, were found to have homologs existing as independent proteins in one of the 50 genomes analyzed. Independent unimodular homologs within *E. coli *were detected for only 57% of the modules (data not shown). A list of the major types of multimodular proteins is shown in Table 3.

**Table 2:** Features of multimodular *E. coli *proteins:

	No. Modules
109 multimodular proteins	226
	
101 bimodular proteins	202
8 trimodular proteins	24
	
with identity to unfused orthologs	203
without identity to unfused orthologs	23
	
known function	151
putative function	66
unknown function	9
	
type of protein^1^:	
enzyme	97
transport protein	85
regulatory protein	26
other	18

^1 ^includes putative assignments

**Table 3:** Types of multimodular proteins.

**Protein type^1^**	**Protein names^2^**
Enzyme	Aas, AdhE, AegA, ArgA, ArnA, CysG, Dfp, DgoA, DsbD, FadB, FadJ, FtsY, GlcE, GlmU, GlnE, Gsp, HisB, HisI, HldE, HmpA, MaeB, MetL, MrcA, MrcB^3^, NifJ^3^, PaaZ, PbpC, PheA, PolA, PurH, PutA, RbbA^3^, RibD, Rne^3^, ThrA, TrpC, TrpD, TyrA, YdiF, YfiQ, YgfN, YgfT, YjiR

Transport protein	AlsA, AraG, CydC, CydD, DhaH, Ego, FeoB, FhuB, FruA, FruB, FrvB, HrsA^3^, KefA, MacB, MalK, MalX, ManX, MdlA, MdlB, MglA, ModF, MsbA, MtlA, NagE^3^, PtsA, PtsG, PtsP, RbsA, ThiP, Uup, XylG, YbhF, YbiT, YddA, YejF, YheS, YjjK, YliA, YnjC, YojI, YpdD^3^, YphE

Regulatory protein	Ada, Aer, ArcB, AtoC, BarA, BglF, CheA, CheB, EvgS, GlnG, KdpD, MalT, RcsC, TorS, YdcR, YfhA, YieN, ZraR

Other	InfB, MukB^3^, RnfC, YegH, YfcK, YoaE

^1^Gene type includes known and putative functions.

^2^Protein names derived from gene names.

^3^Genes encoding three modules.

• Many of the multimodular enzymes function in the biosynthesis or degradation of compounds (amino acids, cofactors, peptidoglycan and fatty acids).

• The majority of the multimodular transport proteins encode fusions of components of the ABC superfamily transporters (ATP-binding and membrane component). Also, fusions of the PTS proteins were detected in different combinations. Thirteen proteins contained two or more PTS components, including Hpr, enzymes I, IIA, IIB, or IIC.

• Among the multimodular regulatory proteins, two-thirds were part of two-component regulatory systems and contained histidine kinases fused to response regulators. Seldom were known domain subdivisions within these modules detected by the rules we applied.

While the fraction of enzymes (39%) is similar to the fraction of enzymes encoded in the genome as a whole (36%), the proportion of multimodular transport proteins (38%) and regulatory proteins (17%) were higher than their proportion genome wide (14% and 8% respectively). The over-representation in transporters and regulators is a reflection of the level of gene duplication seen for these proteins. Large paralogous groups are detected for some of the ABC transporter protein subunits and for components of the two-component regulators.

### Pairwise similarity of *E. coli *single modules

All unimodular proteins, including the modules obtained from multimodular proteins, were tested pairwise for sequence similarity. Matching all single module *E. coli *proteins to each other using the AllAllDb algorithm of the Darwin package, we collected all aligned pairs with a similarity score of less than or equal to 200 PAM units, with an alignment of at least 83 residues. Altogether 9,626 unique pairs met these criteria (data available at GenProtEC [16]).

### Paralogous groups of *E. coli *protein modules

We used the data on pairwise similarity to assemble groups of proteins of similar sequence that were unlike other proteins in the cell. Besides the PAM less than 200 and alignment length of at least 83 residues, two additional requirements were imposed; that more than 45% of each protein in each pair be aligned, and that a module could not belong to more than one group. A transitive clustering process was used to form the sequence-similar groups [9]. This grouping method requires only that each member of the group have sequence similarity to at least one other member of the group and does not require a detectable similarity among all the members of a group. Both closely related groups and groups with more divergent proteins were found.

We identified 490 sequence-similar or paralogous groups in *E. coli *' [see Additional file 2 for a complete list of the sequence-similar *E. coli *groups and their members]'. Altogether 1946 unimodular proteins belonged to one of the groups. Modules from 94 of the multimodular proteins were present in 61 of the groups. Table 4 shows the power law type of distribution of the number of members in the groups, smaller groups being more abundant than large ones. There were 279 groups of two proteins, and only 10 % of the groups had 7 or more members. As shown in Table 5, the smaller groups tended to be tight groups in which the majority of sequences were related by our criteria to all or most others in the group. Larger groups were more divergent with a minority of members related to all others. At group size 8 and above, no members have the property of relating to all others.

**Table 4:** Size distribution of paralogous groups.

**Group size**	**No. Groups**
2	279
3	91
4	32
5	31
6	6
7	18
8	7
9	2
10	2
11	3
12	1
13	2
14	2
18	2
20	1
21	1
22	2
24	1
30	2
40	1
43	1
46	1
51	1
92	1

**Table 5:** Sequence relationships within paralogous groups.

**Group size**	**No. Groups**	**All See All**	**All See Some**
3	92	56	36
4	32	21	11

5	31	7	24
6	6	0	6
7	18	2	16

The largest groups of paralogous enzymes, transport proteins and regulatory proteins are shown in Table 6, 7 and 8, respectively. While enzymes represent the largest gene product type in *E. coli *with known or predicted function, they tend to be present in smaller paralogous groups as compared to the transporters and regulators. Among the larger groups the oxidoreductases and the subunits of oxidoreductases are most common, making up 8 of the top 20 enzyme groups (Table 6).

**Table 6:** Paralogous enzyme groups in *E. coli*.

**No. Members**	**Group function**
20	oxidoreductase, Fe-S-binding
18	oxidoreductase, NAD(P)-binding
18	oxidoreductase^1^, NAD(P)-binding
13	aldehyde oxidoreductase, NAD(P)-binding
13	oxidoreductase, FAD/NAD(P)-binding
11	sugar kinase
10	terminal oxidoreductase, subunit
9	aldo-keto oxidoreductase, NAD(P)-binding
8	phosphatase
8	nucleoside diphosphate (Nudix) hydrolase
8	acyl-CoA ligase
7	glutathione S-transferase
7	RNA helicase, ATP-binding
7	sugar epimerase/dehydratase, NAD(P)-binding
7	alcohol oxidoreductase
7	acyltransferase
7	aminotransferase, PLP-binding
7	decarboxylase, TPP-binding
7	crotonase
7	acyltransferase

^1^Contains GroES-like structural domain (SCOP sf50129).

**Table 7:** Paralogous transport protein groups in *E. coli*

**No. Members**	**Group function**
92	ABC superfamily transport protein, ATP-binding component
51	ABC superfamily transport protein, membrane component
40	MFS family transport protein
24	ABC superfamily transport protein, periplasmic binding component/ transcriptional regulator (GalI/LacR family)/
22	APC family transport protein
12	ABC superfamily transport protein, membrane component
11	PTS family transport protein, enzyme IIA
9	ABC superfamily transport protein, periplasmic binding component
8	ABC superfamily transport protein, periplasmic binding component
7	GntP family transport protein
7	RND family transport protein
7	ABC superfamily transport protein, membrane component
5	HAAP family transport protein
5	PTS family transport protein, enzyme IIB
5	PTS family transport protein, enzyme I
5	GPH family transport protein
5	NCS2 family transport protein
5	HAAP family transport protein
5	transport protein
5	PTS family enzyme IIC
5	RhtB family transport protein
5	outer membrane porin

**Table 8:** Paralogous regulatory protein groups in *E. coli.*

**No. Members**	**Group function**
46	LuxR/UhpA or OmpR family transcriptional response regulator of two-component regulatory system
43	LysR family transcriptional regulator
30	GntR or DeoR family transcriptional regulator
22	sensory histidine kinase in two-component regulatory system
14	sigma54 activator protein, enhancer binding protein
14	AraC/XylS family transcriptional regulator
7	ROK family transcriptional regulator/sugar kinase
7	IclR family transcriptional regulator
5	methyl-accepting chemotaxis protein
5	MerR family transcriptional regulator
4	DNA-binding regulatory protein
3	AraC/XylS family transcriptional regulator
3	MarR family transcriptional reguator
3	AsnC family transcriptional regulator

ATP-binding components of the ABC superfamily of transport proteins are highly conserved and make up the overall largest paralogous group in *E. coli *(Table 7). The other two components of the ABC superfamily transporters are less conserved with membrane components in groups of 52 or less and periplasmic binding components in groups of 9 or less. Components of the PTS system; enzyme IIA, IIB, IIC and I also formed sequence similar groups. One of the groups classified as a group of transporter proteins actually contains both transport proteins (periplasmic binding components of the ABC superfamily) and regulatory proteins (transcriptional regulators of the GalR/LacI family). These two functional types are sequence related, and all of the proteins contain a common structural domain (SCOP sf53822) for the binding of small molecules [25,26]. The difference lies in the presence or absence of a DNA-binding domain.

Response regulators of two-component regulatory systems make up the largest group of regulatory proteins in *E. coli *(Table 8). Sensory histidine kinases of two-component regulatory systems and the sigma54 activating proteins also constitute paralogous groups. A group almost equal in size to the response regulators is the LysR-family of transcriptional regulators. Other large groups of transcriptional regulators are also present.

### Cross genome comparisons of paralogous groups

In addition to using paralogous groups for intra-genomic analyses, the groups were also used in cross genome comparisons (see Table 9). The sizes of selected sequence related groups are shown for three bacteria, the closely related enterics *E. coli *and *Salmonella enterica *serovar Typhimurium and the more distantly related organism *Bacillus subtilis*. The sizes of the groups in the closely related bacteria are similar, whereas there are differences in relation to *B. subtilis*, a gram positive soil organism. For instance, the largest *E. coli *enzyme group containing Fe-S-binding oxidoreductases was represented by only one homolog in the *B. subtilis *genome. However, *B. subtilis *encodes for 31 oxidoreductases homologous to the group of 18 NAD(P)-binding oxidoreductases of *E. coli*. The number of homologous sugar kinases, respiratory reductase subunits, and nucleoside diphosphate (Nudix) hydrolases appeared overall to be lower in *B. subtilis*.

**Table 9:** Cross genome comparisons of enzyme groups.

**Ec^1^**	**So^2^**	**Bs^3^**	**Group function**
20	18	1	oxidoreductase, Fe-S-binding
18	14	31	oxidoreductase, NAD(P)-binding
18	13	10	oxidoreductase^4^, NAD(P)-binding
13	13	11	aldehyde dehydrogenase, NAD(P)-binding
13	11	13	oxidoreductase, FAD/NAD(P)-binding
11	16	6	sugar kinase
10	13	5	respiratory reductase, alpha subunit
9	8	8	aldo-keto reductase, NAD(P)-binding
8	7	5	phosphatase
8	8	2	nucleoside diphosphate (Nudix) hydrolase

^1^No. proteins in *Escherichia coli *paralogous group

^2^No. sequence matches for *E. coli *paralogous group in *Salmonella typhimurium *LT2

^3^No. sequence matches for *E. coli *paralogous group in *Bacillus subtilis*

^4^Contains GroES-like structural domain (SCOP sf50129).

## Discussion

### Protein modules vs. protein domains

We have attempted to enumerate fused genes in *E. coli *in earlier work. Although we recognized the difference between independent proteins with complete function, called modules [9], as opposed to parts of proteins such as motifs and domains, we were not successful in our most recent effort in collecting only complete proteins to the exclusion of domains [15,27]. In earlier work we depended on size as a criterion to eliminate domains, but we know now some domains are large and overlap the lower range of sizes of independent proteins [28]. We also limited our previous studies to alignments between *E. coli *proteins. In this report we make use of information from 50 genomes to detect complete and independent protein homologs for the components of the fused *E. coli *proteins. The need to make use of additional genome sequences is supported by the fact that only 57% of the modules in fused *E. coli *proteins had unfused homologs within the *E. coli *genome while 90% had homologs among the 50 genomes. This result suggests that additional fused *E. coli *proteins might be detected in the future with more available genome sequences.

The overall effect of changing the methodology has been to reduce the numbers of multimodular proteins identified in *E. coli *K-12. As a result of reducing the number of fused proteins, the number of paralogous protein groups was also reduced. The grouping process is based on similarity between the sequences hence many parts of the same proteins remained together in the new groups.

### The effects of multimodular proteins on annotation of genes

For many years we have known that the *E. coli *contained fused genes and groups of sequence-similar proteins [19]. Today with the sequence of the entire genome and that of many other microbial genomes, we can quantify the gene fusions in *E. coli *and apply this information to generate paralogous groups. Even though we find that multimodular proteins are a minor fraction, 2.5%, of the proteins in *E. coli *K-12 MG1655, they significantly affect the annotation of related genes and the ability to define paralogous genes within a genome.

**Figure 3:**
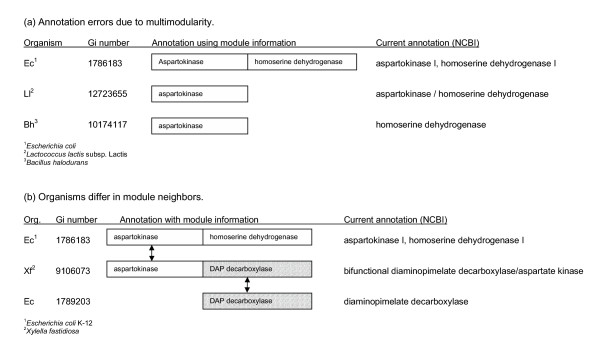
Annotation and composition of multimodular proteins. **(a) **Annotation is complicated by multimodular proteins. An *E. coli *protein (gi1786183) contains two modules, an N-terminal aspartokinase and a C-terminal homoserine dehydrogenase. Two single module proteins from *L. lactis *and *B. halodurans *(gi12723655 and gi10174117) align to the N-terminal aspartokinase module of the *E. coli *protein. Based on the sequence alignments, both of these proteins should be annotated as aspartokinases. However, errors are seen in the annotation of the *L. lactis *and *B. halodurans *proteins stemming from transfer of functions between multimodular proteins and partially aligned sequences without taking into account the alignment regions. **(b) **Different combinations of modules are seen in multimodular proteins of different organisms. While aspartokinase is fused to homoserine dehydrogenase in *E. coli *it is fused to DAP decarboxylase in *X. fastidiosa*. In both organisms the fusions are between enzymes of metabolic pathways, threonine biosynthesis for *E. coli *and lysine biosynthesis in *X. fastidiosa*.

Examples of the types of errors arising in the annotation of fused proteins are shown in Figure 3a. The multimodular protein ThrA (gi1786183) encodes an aspartokinase in the N-terminal module (aa 1–461) and a homoserine dehydrogenase in the C-terminal module (aa 464–820). A sequence similar protein from *Lactococcus lactis*, gi12723655, aligning only to the N-terminal module is erroneously annotated as having both aspartokinase and homoserine dehydrogenase activities. The correct annotation should be aspartokinase. In a second example, a protein from *Bacillus halodurans*, gi10174117, aligns to the aspartokinase module of ThrA but is described as homoserine dehydrogenase. The correct assignment should be aspartokinase.

As shown in Figure 3b, different genes are sometimes fused to the same gene in different organisms. In *E. coli *an aspartokinase is fused to a homoserine dehydrogenase (gi1766183), while in *Xylella fastidiosa*, an aspartokinase is fused to a diaminopimelate decarboxylase (gi9106073). One needs to be alert to partial alignments. In this case, the annotation is correct for both activities of the Xylella protein, although the description does not follow the convention of stating the N-terminal activity first, raising the potential for misidentification of the activity of a partial homolog.

### Generality of gene fusions and remedies

The details of gene duplication and divergence and of gene fusions have followed different courses in separate lines of descent of bacteria. The fusions of different gene partners to aspartokinase in *E. coli *and *X. fastidiosa *connected proteins acting in the same pathway. However, the pathways are different for the two organisms, threonine biosynthesis for *E. coli *and lysine biosynthesis in *X. fastidiosa*. Fusions of genes in a pathway have long been known and also the fusions of different genes in different organisms. In the tryptophan biosynthesis pathway of *E. coli *both the *trp*C gene (formerly trpC(F)) and the *trp*D gene (formerly *trp*G(D)) encode two enzymes as indicated in their former names. In contrast *Rhizobium meliloti *has a fusion between the *trp*E and *trp*G genes, *trp*E(G) [29]. Such differences not taken into account in annotation have generated errors in assignment of activities in some of the tryptophan synthesis proteins in a number of organisms. The variability in gene fusions among bacteria means that definition of multimodular proteins cannot be transferred from one organism to another, but must be worked out by analyzing the partial homology patterns with smaller independent proteins found in other organisms.

To promote awareness of fused proteins, databases should list such proteins with their separate component activities and the approximate locations of these; either by start and end residues, or by module location (N-terminal, C-terminal, or Middle for proteins with >2 modules). Such a format has been implemented in GenProtEC [16]. When analyzing protein sequence alignments, one should make use of information on the alignment lengths and on the percent of each sequence that is involved in the alignment. Such information may hold clues to detecting fused proteins.

### Properties of paralogous groups of *E. coli*

**Figure 4:**
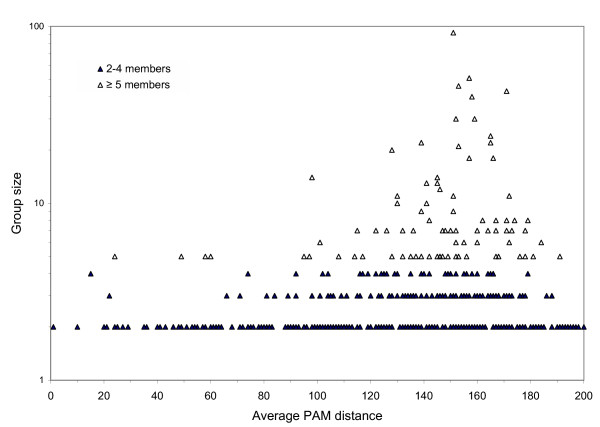
Sequence similarity of *E. coli *paralogous protein groups versus the group size. Protein sequences were aligned by the AllAllDb program of Darwin. Multimodular proteins were separated into modules (independent functional units) prior to the Darwin analysis. Alignments with similarities of ≤ 200 PAM units over 83 amino acids and where >45% of the length of both proteins in the pair were aligned were used to generate protein groups. The average PAM distances for the protein pairs in the smaller groups having 2–4 members (▲) and in the larger groups of ≥ 5 members (△) are shown. The smaller groups are more abundant and show a wide range of similarities. The larger groups appear to be more divergent with higher average PAM values clustering around PAM 150.

Groups of unimodular *E. coli *proteins similar in sequence vary in size from two (simple pairs) up to 92 members (Table 4). From pairs to groups of 8, the number of paralogous groups follows a power law. Above size 8, most sizes are represented by just one or two groups. For the smallest groups, two to four members, the degree of sequence similarity (PAM scores) tend to range widely (Figure 4). As the groups are larger, a clear distribution around PAM 150 emerges. Perhaps the larger groups are ones whose success is reflected in many duplication events over time with a retained function if the sequence drift is held to the range 100 to 200 PAM units. It appears that choosing 200 PAM as the upper ceiling has not eliminated an important number of groups with highly diverged members. Also, the broad range of degree of relatedness among members of paralogous groups (Table 5, Figure 4) suggests that some types of proteins diverge further than others. The cluser around PAM 150 is populated by large successful paralogous groups, some of which are closely related in catalytic function while others have diverged to more distantly related activities.

The largest paralogous groups are transporters and regulators (Tables 7 &8). Paralogous groups of enzymes tend to be smaller (Table 6). The largest enzyme classes tend to be oxidoreductases or subunits of oxidoreductases, and the relationships among members of these groups point in the direction of shared binding capacities accounting for the sequence relatedness, e.g. Fe-S clusters. In earlier work we found that some sequence related enzymes are alike in their ligand-binding characteristics, others are alike in mechanism of the catalytic action [30]. Both types of shared properties are seen in Table 6.

The ABC transporters have been a successful formula in bacterial evolution. The ATP-binding subunits maintain detectable sequence similarity. More divergent are the membrane subunits, and least similar are the periplasmic ligand-binding subunits, perhaps understandably divergent as their binding specificities for each transported compound will differ with the properties of the compounds [31]. One of the groups of periplasmic binding components also contains sequence related transcriptional regulators of the GalR/LacI family, agreeing with previous reports [25,26]. The major difference between these two functions is the presence or absence of a DNA-binding domain. According to Fukami-Kobayashi et al. [26], the regulators in this group are believed to have arisen by the fusion of a DNA binding domain to an ancestral periplasmic binding protein. The substrate specificity is thought to have evolved subsequently. Only a few of the transporters and regulators in this group bind the same substrates; galactose (MglB and GalR), ribose (RbsR and RbsB) and xylose (XylF and XylR).

Among the regulator groups (Table 8), the class of two-component regulators is large. The two major activities of sensory histidine kinase and response regulators separate by the rules for grouping modules, but their known internal structures do not emerge. Many other groups are different kinds of transcriptional regulators. Another example of different functions related by sequence has been reported for a class of repressors and kinases, the ROK family [32]. In this case the two different functions are sequence related via their sugar-binding domains and differ in their DNA-binding or kinase activity.

### Cross genome comparisons

Examining comparable paralogous groups among organisms may provide insight into functional and physiological differences among organisms. Illustration of the possibilities is shown in Table 9 where the sizes of comparable paralogous groups are shown for the closely related enteric bacteria *E. coli *and *S. enterica *serovar Typhimurium and the distant gram positive soil organism *B. subtilis*. Major difference is seen for one category of oxidoreductases. The largest enzyme group in *E. coli *contains 20 FeS-binding proteins whereas the *B. subtilis *genome has only one protein of this type. Members of the *E. coli *group include subunits of formate dehydrogenases, hydrogenases 3 and 4, DMSO reductase, and a NADH dehydrogenase. The presence of elements of the formate hydrogen lyase system and of the DMSO reductase in *E. coli *but not *B. subtilis *illustrates information on metabolic differences that emerges from such cross-genome comparisons. *B. subtilis *does not have the diverse anaerobic respiratory capability of *E. coli *and *S. enterica*. Duplication and divergence of this common ancestral gene seems to have taken a different course in the two bacterial lineages.

In another example, *B. subtilis *has made use of one enzyme type to a greater extent than the two enteric organisms. The number of one of the types of NAD(P)-binding oxidoreductases is much larger in *B. subtilis *(31 proteins) than in the enterics (18 proteins). The *B. subtilis *enzymes in this group are fatty acid biosynthesis enzymes, agreeing with the known fact that this organism synthesizes a greater variety of fatty acids and has dedicated more of its proteome towards diversifying its fatty acid biosynthetic capabilities [33,34]. Thus sequence similar groups may be used in comparative analysis between genomes, highlighting areas where genetic resources have been expanded, pointing up metabolic differences between organisms.

## Conclusion

• Proteins encoded by fused genes, multimodular proteins, require special attention in genome analysis. Such multimodular proteins contain two or more functional components that are located at separate parts of the protein and that may exist as independent proteins in other genomes. Annotation of the multimodular proteins should include the separate functions and their corresponding locations in the gene product. This will improve transfer of function between the fused proteins and sequences matching their entire length or only the length of one of their module components. Current annotation errors involving fused genes can be remedied by introducing this approach.

• The identification of multimodular proteins in *E. coli *was improved by making use of sequence information from 50 genomes to detect alignments between the fused proteins and smaller, un-fused homologs corresponding to the component modules. The more common multidomain proteins, proteins containing fused sequence domains or motifs that together make up one overall function, were not detected as multimodular proteins by this approach. As a result the current number of fused *E. coli *proteins was reduced to 109 proteins with 8 containing three modules and 101 containing two modules. The multimodular *E. coli *proteins consist mainly of enzymes, regulators and transport proteins. Their component modules are often not related by sequence but many are related in that they function in a common pathway or cell role. Components of fused genes appear to vary from genome to genome hence complicating their detection and function assignment.

• Multimodular proteins are different from multifunctional proteins in that the latter catalyze more than one reaction in the same region of the protein.

• The generation of paralogous or sequence related groups is improved when the modules of multimodular proteins are separated and treated as independent proteins for the grouping process. 490 groups of sequence related *E. coli *proteins ranging in size from 2 to 92 were generated from the new module data. The smaller groups range widely in degree of relatedness while the larger groups have diverged from one another to about the same extent. Transport proteins and regulatory proteins were found in the larger groups while enzyme groups tended to have fewer members.

• Over half of the *E. coli *proteins belong to paralogous groups, reflecting the prominent role of duplication and divergence in the evolution of the genome. The number and sizes of paralogous groups reflect the distinctiveness of the organisms and they can be used in cross genome comparisons.

## Methods

### Sequence sources

Protein coding sequences were obtained from GenBank and included the following genomes: *Aquifex aeolicus*, (AE000657); *Archaeoglobus fulgidus*, (AE000782); *Aeropyrum pernix*, (BA000002); *Agrobacterium tumefaciens*, (AE007869/AE007870); *Borrelia burgdorferi*, (AE000783); *Bacillus halodurans*, (BA000004); *Bacillus subtilis*, (AL009126); *Buchnera *sp. APS, (BA000003); *Campylobacter jejuni*, (AL111168); *Clostridium acetobutylicum*, (AE001437); *Chlamydia muridarum*, (AE002160); *Chlamydophila pneumoniae *CWL029, (AE001363); *Deinococcus radiodurans*, (AE000513/AE001823); *Escherichia coli *K-12, (U00096); *Escherichia coli *O157:H7 EDL933, (AE005174); *Escherichia coli *O157:H7, (BA000007); *Haemophilus influenzae*, (L42023); *Helicobacter pylori *26695, (AE000511); *Halobacterium *sp. NRC-1, (AE004437); *Lactococcus lactis *subsp.*lactis*, (AE005176); *Mycobacterium leprae*, (AL450380); *Mycoplasma genitalium*, (L43967); *Mycobacterium tuberculosis *H37Rv, (AL123456); *Methanococcus jannaschii*, (LL77117); *Mesorhizobium loti*, (BA000012); *Mycoplasma pneumoniae*, (U00089); *Mycoplasma pulmonis*, (AL445566); *Methanobacterium thermoautotrophicum*, (AE000666); *Neisseria meningitidis *MC58, (AE002098); *Pseudomonas aeruginosa*, (AE004091); *Pyrococcus horikoshii*, (BA000001); *Pasteurella multocida*, (AE004439); *Pyrococcus abyssi*, (AL096836); *Rickettsia prowazekii*, (AJ235269); *Salmonella enterica *subsp. *enterica *serovar Typhi, (NC_003198); *Salmonella typhimurium *LT2, (AE006468); *Shewanella oneidensis *MR-1, (NC004347); *Sinorhizobium meliloti*, (AL591688); *Staphylococcus aureus *subsp.*aureus *Mu50, (BA000017); *Streptococcus pneumoniae *TIGR4, (AE005672); *Streptococcus pyogenes *M1 GAS, (AE004092); *Sulfolobus solfataricus*, (AE006641); *Synechocystis *PCC6803, (AB001339); *Thermoplasma volcanium*, (BA000011); *Thermotoga maritima*, (AE000512); *Treponema pallidum*, (AE000520); *Ureaplasma urealyticum*, (AF222894); *Vibrio cholerae*, (AE003852/EC003853); *Xylella fastidiosa *9a5c, (AE003849); *Yersinia pestis*, (AL590842).

### Analysis of protein sequence similarities

Pairwise sequence alignments and scores were generated using the AllAllDb program of Darwin (Data Analysis and Retrieval With Indexed Nucleotide/peptide sequence package), version 2.0, developed at the ETHZ in Zurich [35]. Maximum likelihood alignments are generated with an initial global alignment by dynamic programming [36-38] followed by dynamic local alignments [39]. A single scoring matrix is used for these steps. After the initial alignment, the scoring matrix is adjusted to fit the approximate distance between each protein pair to produce the minimum PAM value. PAM units are defined as the numbers of point mutations per 100 residues [37]. The final report includes PAM distances and variances.

For the work reported here, sequence pairs were collected that had alignment lengths of at least 83 amino acids and distances of 200 PAM units or less. We chose the length requirement of 83 residues as it improves the significance of the sequence alignments for the more distantly related protein pairs [40]. The requirement for at least 83 residues also avoids a class of commonly occurring protein domains smaller than 83 residues that appear widely in many otherwise unrelated proteins (such as small binding sites for a type of substrate, cofactor, or regulator). In addition for this study we removed proteins directly involved in horizontal gene transfer (IS proteins, transposases, and known prophage components) from the dataset.

### Identification of multimodular proteins

Proteins encoded by fused genes were identified from the *E. coli *literature and from unequal sequence alignments. The literature was searched for *E. coli *proteins with more than one function encoded at separate parts of the protein. The locations of the alignment regions in the proteins were analyzed for orthologous and paralogous protein pairs. We identified proteins with two or more non-overlapping alignment regions where each region aligned separately to smaller homologs. Figure 1a illustrates the alignment of two unfused proteins with parts of a fused protein. Multimodular proteins so identified were separated into independent modules. Using the pairwise data, start and end positions of the modules were estimated from the many alignment regions and were set to cover as much of the sequence as possible, not only the most conserved regions of all the alignments. No overlap was allowed between any adjacent modules.

### Generation of internal sequence similar groups (paralogs)

The sum of the separated modules from the multimodular proteins and the naturally occurring unimodular proteins of *E. coli *were aligned against themselves. Protein pairs aligning with >45% of the length of the peptides were used in a transitive grouping process as previously described [15]. The transitive nature of the process ensures sequence similarity to at least one member of the group and does not require all members of the group to have detectable similarity to one another. This type of clustering allows for more divergent sequences to be grouped. The restriction of PAM value to no more than 200 prevents groups from expanding beyond significant similarity.

## Authors' contributions

MS designed the study, performed the sequence analysis, and participated in the data analysis and in writing the manuscript. MR participated in the data analysis and in writing the manuscript.

## Supplementary Material

Additional File 1 Multimodular *E. coli *proteins. The table contains a complete list of the multimodular proteins in *E. coli*. Each module is described by its Gene name, Module Id, Module Start and End positions, Gene type, and Module Product. Click here for file

Additional File 2 E. coli paralogous groups and their members. The table contains a complete list of the paralogous protein groups in *E. coli*. The members of the 409 paralogous groups are indicated by their Group Membership, Module Id, Module Start and End Position, Module Product. Click here for file

## References

[B1] Glasner JD, Liss P, Plunkett G, Darling A, Prasad T, Rusch M, Byrnes A, Gilson M, Biehl B, Blattner FR (2003). ASAP, a systematic annotation package for community analysis of genomes. Nucleic Acids Res.

[B2] Chaudhuri RR, Khan AM, Pallen MJ (2004). coliBASE: an online database for Escherichia coli, Shigella and Salmonella comparative genomics. Nucleic Acids Res.

[B3] Sundararaj S, Guo A, Habibi-Nazhad B, Rouani M, Stothard P, Ellison M, Wishart DS (2004). The CyberCell Database (CCDB): a comprehensive, self-updating, relational database to coordinate and facilitate in silico modeling of Escherichia coli. Nucleic Acids Res.

[B4] Thomas GH (1999). Completing the E. coli proteome: a database of gene products characterised since the completion of the genome sequence. Bioinformatics.

[B5] Karp PD, Riley M, Saier M, Paulsen IT, Collado-Vides J, Paley SM, Pellegrini-Toole A, Bonavides C, Gama-Castro S (2002). The EcoCyc Database. Nucleic Acids Research (Online).

[B6] Serres MH, Goswami S, Riley M (2004). GenProtEC: an updated and improved analysis of functions of Escherichia coli K-12 proteins. Nucleic Acids Res.

[B7] Salgado H, Gama-Castro S, Martinez-Antonio A, Diaz-Peredo E, Sanchez-Solano F, Peralta-Gil M, Garcia-Alonso D, Jimenez-Jacinto V, Santos-Zavaleta A, Bonavides-Martinez C (2004). RegulonDB (version 4.0): transcriptional regulation, operon organization and growth conditions in Escherichia coli K-12. Nucleic Acids Res.

[B8] Riley M (2004). Workshop on Annotation of Escherichia coli K-12. ASM News 70[1], 2-2 Ref Type: Magazine Article.

[B9] Riley M, Labedan B (1997). Protein evolution viewed through Escherichia coli protein sequences: introducing the notion of a structural segment of homology, the module. J Mol Biol.

[B10] Bateman A, Coin L, Durbin R, Finn RD, Hollich V, Griffiths-Jones S, Khanna A, Marshall M, Moxon S, Sonnhammer EL (2004). The Pfam protein families database. Nucleic Acids Res.

[B11] Madera M, Vogel C, Kummerfeld SK, Chothia C, Gough J (2004). The SUPERFAMILY database in 2004: additions and improvements. Nucleic Acids Res.

[B12] Vogel C, Bashton M, Kerrison ND, Chothia C, Teichmann SA (2004). Structure, function and evolution of multidomain proteins. Curr Opin Struct Biol.

[B13] Galperin MY, Koonin EV (1998). Sources of systematic error in functional annotation of genomes: domain rearrangement, non-orthologous gene displacement and operon disruption. In Silico Biol.

[B14] Gilks WR, Audit B, De Angelis D, Tsoka S, Ouzounis CA (2002). Modeling the percolation of annotation errors in a database of protein sequences. Bioinformatics.

[B15] Liang P, Labedan B, Riley M (2002). Physiological genomics of Escherichia coli protein families. Physiol Genomics.

[B16] El Ghachi M, Bouhss A, Blanot D, Mengin-Lecreulx D (2004). The bacA gene of Escherichia coli encodes an undecaprenyl pyrophosphate phosphatase activity. J Biol Chem.

[B17] Yang SY, Schulz H (1983). The large subunit of the fatty acid oxidation complex from Escherichia coli is a multifunctional polypeptide. Evidence for the existence of a fatty acid oxidation operon (fad AB) in Escherichia coli. J Biol Chem.

[B18] Gerlt JA, Babbitt PC (2001). Divergent evolution of enzymatic function: mechanistically diverse superfamilies and functionally distinct suprafamilies. Annu Rev Biochem.

[B19] Labedan B, Riley M (1995). Widespread protein sequence similarities: Origins of Escherichia coli genes. J Bacteriol.

[B20] Serres MH, Gopal S, Nahum LA, Liang P, Gaasterland T, Riley M (2001). A functional update of the Escherichia coli K-12 genome. Genome Biology (Online).

[B21] Jaggi R, van Heeswijk WC, Westerhoff HV, Ollis DL, Vasudevan SG (1997). The two opposing activities of adenylyl transferase reside in distinct homologous domains, with intramolecular signal transduction. EMBO J.

[B22] Dautry-Varsat A, Cohen GN (1977). Proteolysis of the bifunctional methionine-repressible aspartokinase II-homoserine dehydrogenase II of Escherichia coli K12. Production of an active homoserine dehydrogenase fragment. J Biol Chem.

[B23] Saint-Girons I, Margarita D (1978). Fine structure analysis of the threonine operon in Escherichia coli K-12. Mol Gen Genet.

[B24] Maruya A, O'Connor MJ, Backman K (1987). Genetic separability of the chorismate mutase and prephenate dehydrogenase components of the Escherichia coli tyrA gene product. J Bacteriol.

[B25] Vartak NB, Reizer J, Reizer A, Gripp JT, Groisman EA, Wu LF, Tomich JM, Saier MH (1991). Sequence and evolution of the FruR protein of Salmonella typhimurium: a pleiotropic transcriptional regulatory protein possessing both activator and repressor functions which is homologous to the periplasmic ribose-binding protein. Res Microbiol.

[B26] Fukami-Kobayashi K, Tateno Y, Nishikawa K (2003). Parallel evolution of ligand specificity between LacI/GalR family repressors and periplasmic sugar-binding proteins. Mol Biol Evol.

[B27] Liang P, Riley M (2001). A comparative genomics approach for studying ancestral proteins and evolution. Advances in Applied Microbiology.

[B28] Gough J, Karplus K, Hughey R, Chothia C (2001). Assignment of homology to genome sequences using a library of hidden Markov models that represent all proteins of known structure. J Mol Biol.

[B29] Crawford IP (1989). Evolution of a biosynthetic pathway: the tryptophan paradigm. Annu Rev Microbiol.

[B30] Nahum LA, Riley M (2001). Divergence of function in sequence-related groups of Escherichia coli proteins. Genome Research.

[B31] Higgins CF (2001). ABC transporters: physiology, structure and mechanism – an overview. Research in Microbiology.

[B32] Titgemeyer F, Reizer J, Reizer A, Saier MH (1994). Evolutionary relationships between sugar kinases and transcriptional repressors in bacteria. Microbiology.

[B33] Fujita Y, Ramaley R, Freese E (1977). Location and properties of glucose dehydrogenase in sporulating cells and spores of Bacillus subtilis. J Bacteriol.

[B34] Piggot PJ, Losick R, Sonenshein AL, Hoch JA, Losick R (2002). Sporulation Genes and Intercompartmental Regulation. Bacillus subtilis and its closest relatives from genes to cells.

[B35] Gonnet GH, Hallett MT, Korostensky C, Bernardin L (2000). Darwin v. 2.0: an interpreted computer language for the biosciences. Bioinformatics.

[B36] Needleman SB, Wunsch CD (1970). A general method applicable to the search for similarities in the amino acid sequence of two proteins. J Mol Biol.

[B37] Schwartz RM, Dayhoff MO, Dayhoff MO (1978). Atlas of Protein Sequence and Structure.

[B38] Gonnet GH, Korostensky C, Benner S (2000). Evaluation measures of multiple sequence alignments. J Comput Biol.

[B39] Smith TF, Waterman MS (1981). Identification of common molecular subsequences. J Mol Biol.

[B40] Altschul SF (1991). Amino acid substitution matrices from an information theoretic perspective. J Mol Biol.

