# Supplementary material for: Gene fusions and gene duplications: relevance to genomic annotation and functional analysis
Source: BMC Genomics. 2005 Mar 9;6:33. doi: 10.1186/1471-2164-6-33 (PMC555942; doi:10.1186/1471-2164-6-33)
Supplement: Additional File 1 — Multimodular E. coli proteins. The table contains a complete list of the multimodular proteins in E. coli. Each module is described by its Gene name, Module Id, Module Start and End positions, Gene type, and Module Product. [file 1471-2164-6-33-S1.pdf]

Additional file 1. Multimodular *E. coli* proteins

Gene	Module	Start	End	Gty[1]	Module Product
*thr*A	b0002_1	1	461	e	aspartokinase I, threonine sensitive
*thr*A	b0002_2	464	820	e	homoserine dehydrogenase I, threonine sensitive
*thi*P	b0067_1	1	274	t	thiamin transport protein (ABC superfamily, membrane)
*thi*P	b0067_2	285	536	t	thiamin transport protein (ABC superfamily, membrane)
*mrc*B	b0149_1	1	200	m	membrane module of bifunctional penicillin-binding protein 1b
*mrc*B	b0149_2	204	382	e	glycosyl transferase of penicillin-binding protein 1b
*mrc*B	b0149_3	444	844	e	transpeptidase of penicillin-binding protein 1b
*fhu*B	b0153_1	1	330	t	hydroxamate-dependent iron transport protein (ABC superfamily, membrane)
*fhu*B	b0153_2	331	660	t	hydroxamate-dependent iron transport protein (ABC superfamily, membrane)
*rib*D	b0414_1	1	143	e	diaminohydroxyphosphoribosylaminopyrimidine deaminase
*rib*D	b0414_2	147	366	e	5-amino-6-(5-phosphoribosylamino) uracil reductase
*mdl*A	b0448_1	1	310	pt	putative transport protein, multidrug resistance-like (ABC superfamily, membrane)
*mdl*A	b0448_2	314	590	pt	putative transport protein, multidrug resistance-like (ABC superfamily, ATP_bind)
*mdl*B	b0449_1	1	320	pt	putative transport protein, multidrug resistance-like (ABC superfamily, membrane)
*mdl*B	b0449_2	321	593	pt	putative transport protein, multidrug resistance-like (ABC superfamily, ATP_bind)
*kef*A	b0465_1	1	779	o	unknown function module of mechanosensitive channel
*kef*A	b0465_2	780	1120	t	mechanosensitive channel (MscS family)
*nag*E	b0679_1	1	311	t	PTS family enzyme IIC, N-acetylglucosamine-specific
*nag*E	b0679_2	341	466	t	PTS family enzyme IIB, N-acetylglucosamine-specific
*nag*E	b0679_3	497	648	t	PTS family enzyme IIA, N-acetylglucosamine-specific
*kdp*D	b0695_1	1	373	r	membrane signal domain of sensory histidine kinase
*kdp*D	b0695_2	402	894	e	transmitter domain of sensory histidine kinase
*hrs*A	b0731_1	1	178	t	PTS family enzyme IIA, induction of ompC
*hrs*A	b0731_2	186	454	t	PTS family enzyme IIB, induction of ompC
*hrs*A	b0731_3	456	628	t	PTS family enzyme IIC, induction of ompC
*mod*F	b0760_1	1	260	t	molybdenum transport protein (ABC superfamily, ATP_bind)
*mod*F	b0760_2	261	490	t	molybdenum transport protein (ABC superfamily, ATP_bind)
*ybh*F	b0794_1	1	292	pt	putative transport protein (ABC superfamily, ATP_bind)
*ybh*F	b0794_2	293	583	pt	putative transport protein (ABC superfamily, ATP_bind)
*ybi*T	b0820_1	1	280	pt	putative transport protein (ABC superfamily, ATP_bind)
*ybi*T	b0820_2	281	530	pt	putative transport protein (ABC superfamily, ATP_bind)
*yli*A	b0829_1	1	279	pt	putative oligopeptide transport protein (ABC superfamily, ATP_bind)
*yli*A	b0829_2	281	612	pt	putative oligopeptide transport protein (ABC superfamily, ATP_bind)
macB	b0879_1	1	253	t	macrolide transport protein (ABC superfamily, ATP_bind)
macB	b0879_2	254	648	t	macrolide transport protein (ABC superfamily, membrane)
cydC	b0886_1	1	318	t	cysteine transport (export) protein (ABC superfamily, membrane)
cydC	b0886_2	319	573	t	cysteine transport (export) protein (ABC superfamily, ATP_bind)
cydD	b0887_1	1	318	t	cysteine transport (export) protein (ABC superfamily, membrane)
cydD	b0887_2	319	588	t	cysteine transport (export) protein (ABC superfamily, ATP_bind)
msbA	b0914_1	1	321	t	lipid transport protein, flippase (ABC superfamily, membrane)
msbA	b0914_2	322	582	t	lipid transport protein, flippase (ABC superfamily, ATP_bind)
mukB	b0924_1	1	454	pe	putative nucleotide hydrolase module of kinesin-like cell division protein
mukB	b0924_2	455	783	o	unknown function module of kinesin-like cell division protein
mukB	b0924_3	784	1486	o	unknown function module of kinesin-like cell division protein
uup	b0949_1	1	290	pt	putative transport protein (ABC superfamily, ATP_bind)
uup	b0949_2	292	635	pt	putative transport protein (ABC superfamily, ATP_bind)
*tor*S	b0993_1	1	665	e	histidine kinase of hybrid sensory kinase
*tor*S	b0993_2	669	904	r	response regulator of hybrid sensory kinase
*put*A	b1014_1	1	569	e	bifunctional: transcriptional repressor (N-terminal); proline dehydrogenase, FAD-binding (C-terminal)
*put*A	b1014_2	618	1320	e	pyrroline-5-carboxylate dehydrogenase
rne	b1084_1	1	509	e	endoribonuclease module of RNase E, rRNA processing and mRNA degradation
rne	b1084_2	511	849	e	RNA binding module of RNase E
rne	b1084_3	851	1061	e	RNA degradosome binding module of RNase E
*pts*G	b1101_1	1	330	t	PTS family enzyme IIC, glucose-specific

*pts*G	b1101_2	360	477	t	PTS family enzyme IIB, glucose-specific
*dha*H	b1198_1	1	259	pt	putative PTS family HPr components
*dha*H	b1198_2	260	473	pt	putative PTS family enzyme I
*adh*E	b1241_1	1	400	e	acetaldehyde-CoA dehydrogenase
*adh*E	b1241_2	449	891	e	iron-dependent alcohol dehydrogenase
*trp*C	b1262_1	1	257	e	indole-3-glycerolphosphate synthetase
*trp*C	b1262_2	258	453	e	N-(5-phosphoribosyl)anthranilate isomerase
*trp*D	b1263_1	1	193	e	glutamine amidotransferase
*trp*D	b1263_2	198	531	e	anthranilate phosphoribosyltransferase
nifJ	b1378_1	1	403	o	unknown function module of putative pyruvate-flavodoxin oxidoreductase
nifJ	b1378_2	418	758	o	unknown function module of putative pyruvate-flavodoxin oxidoreductase
nifJ	b1378_3	817	1174	pc	Fe-S binding module of putative pyruvate-flavodoxin oxidoreductase
*paa*Z	b1387_1	1	506	e	aldehyde dehydrogenase
*paa*Z	b1387_2	519	681	e	enoyl CoA dehydtratase
*ydc*R	b1439_1	1	117	pr	putative transcriptional regulator (GntR family)
*ydc*R	b1439_2	118	468	pe	putative amino transferase
*ydd*A	b1496_1	1	355	pt	putative transport protein (ABC superfamily, membrane)
*ydd*A	b1496_2	356	561	pt	putative transport protein (ABC superfamily, ATP_bind)
ego	b1513_1	1	264	pt	putative sugar transport protein (ABC superfamily, ATP_bind)
ego	b1513_2	265	511	pt	putative sugar transport protein (ABC superfamily, ATP_bind)
*mal*X	b1621_1	1	383	t	PTS family enzyme IIC, maltose/glucose-specific
*mal*X	b1621_2	409	530	t	PTS family enzyme IIB, maltose/glucose-specific
*rnf*C	b1629_1	1	448	pc	Fe-S binding module of electron transport protein
*rnf*C	b1629_2	450	740	o	unknown function module of electron transport protein
*ydi*F	b1694_1	1	258	pe	alpha subunit of putative acetyl-CoA:acetoacetyl-CoA transferase
*ydi*F	b1694_2	281	531	pe	beta subunit of putative acetyl-CoA:acetoacetyl-CoA transferase
*ynj*C	b1755_1	1	251	pt	putative transport protein (ABC superfamily, membrane)
*ynj*C	b1755_2	293	496	pt	putative transport protein (ABC superfamily, membrane)
*yoa*E	b1816_1	1	233	pm	putative transmembrane protein
*yoa*E	b1816_2	234	518	pm	putative transmembrane protein
*man*X	b1817_1	1	132	t	PTS family enzyme IIA, mannose-specific
*man*X	b1817_2	162	323	t	PTS family enzyme IIB, mannose-specific
*che*B	b1883_1	1	108	r	response regulator of regulatory protein
*che*B	b1883_2	157	349	e	methylesterase of regulatory protein
*che*A	b1888_1	1	501	e	histidine kinase of sensory histidine kinase
*che*A	b1888_2	502	654	r	binding module of chemotactic sensory histidine kinase
*ara*G	b1900_1	1	267	t	high-affinity L-arabinose transport protein (ABC superfamily, ATP_bind)
*ara*G	b1900_2	268	504	t	high-affinity L-arabinose transport protein (ABC superfamily, ATP_bind)
*his*B	b2022_1	1	167	e	histidinol-phosphatase
*his*B	b2022_2	168	355	e	imidazoleglycerol-phosphate dehydratase
*his*I	b2026_1	1	112	e	phosphoribosyl-AMP cyclohydrolase
*his*I	b2026_2	113	203	e	phosphoribosyl-ATP pyrophosphatase
*yeg*H	b2063_1	1	259	pm	putative transmembrane protein
*yeg*H	b2063_2	268	549	pm	putative transmembrane protein
*mgl*A	b2149_1	1	258	t	galactose (methyl-galactoside) transport protein (ABC superfamily, ATP_bind)
*mgl*A	b2149_2	259	506	t	galactose (methyl-galactoside) transport protein (ABC superfamily, ATP_bind)
*fru*A	b2167_1	1	201	t	PTS family enzyme IIB'B, fructose-specific
*fru*A	b2167_2	228	563	t	PTS family enzyme IIC, fructose-specific
*fru*B	b2169_1	1	149	t	PTS family enzyme IIA, fructose-specific
*fru*B	b2169_2	151	376	t	PTS family enzyme FPr, fructose-specific
*yej*F	b2180_1	1	287	pt	putative oligopeptide transport protein (ABC superfamily, ATP_bind)
*yej*F	b2180_2	288	529	pt	putative oligopeptide transport protein (ABC superfamily, ATP_bind)
*yoj*I	b2211_1	1	306	pt	putative transport proteins (ABC superfamily, membrane)
*yoj*I	b2211_2	307	547	pt	putative transport proteins (ABC superfamily, ATP_bind)
ada	b2213_1	1	183	r	transcriptional regulator of DNA repair
ada	b2213_2	189	354	e	O6-methylguanine-DNA methyltransferase
*rcs*C	b2218_1	1	677	e	histidine kinase of hybrid sensory kinase
*rcs*C	b2218_2	806	933	r	response regulator of hybrid sensory kinase

*ato*C	b2220_1	1	125	r	response regulator
*ato*C	b2220_2	145	461	r	sigma54 interaction module of response regulator (EBP family)
*arn*A	b2255_1	1	305	e	oxidoreductase module of UDP-D-glucuronate dehydrogenase
*arn*A	b2255_2	312	660	pe	putative formyltransferase module of UDP-D-glucuronate dehydrogenase
*yfc*K	b2324_1	1	262	o	unknown function module of conserved protein
*yfc*K	b2324_2	275	688	o	putative FAD/NAD(P)-binding oxidoreductase module of conserved protein
*fad*J	b2341_1	1	255	e	enoyl-CoA hydratase;epimerase;isomerase of anaerobic fatty acid oxidation complex protein
*fad*J	b2341_2	274	714	e	3-hydroxyacyl-CoA dehydrogenase of anaerobic fatty acid oxidation complex protein
evgS	b2370_1	1	935	e	histidine kinase of hybrid sensory kinase
evgS	b2370_2	953	1197	r	response regulator of hybrid sensory histidine kinase
*ypd*D	b2383_1	1	108	pt	putative PTS family Hpr component
*ypd*D	b2383_2	110	688	pt	putative PTS family enzyme I component
*ypd*D	b2383_3	690	831	pt	putative PTS family enzyme IIA component
*mae*B	b2463_1	1	422	pe	putative malic oxidoreductase
*mae*B	b2463_2	423	759	pe	putative phosphotransacetylase
*aeg*A	b2468_1	1	165	pc	Fe-S binding module of putative oxidoreductase
*aeg*A	b2468_2	181	659	pe	NAD/FAD-binding module of putative oxidoreductase
*pbp*C	b2519_1	1	230	e	transglycosylase of penicillin-binding protein 1c
*pbp*C	b2519_2	269	770	e	transpeptidase of penicillin-binding protein 1c
*yph*E	b2547_1	1	269	pt	putative sugar transport protein (ABC superfamily, ATP_bind)
*yph*E	b2547_2	270	503	pt	putative sugar transport protein (ABC superfamily, ATP_bind)
*hmp*A	b2552_1	1	140	e	nitric oxide dioxygenase
*hmp*A	b2552_2	141	396	e	dihydropteridine reductase 2
*yfh*A	b2554_1	1	130	pr	putative response regulator
*yfh*A	b2554_2	132	444	pr	putative sigma54 interaction module of response regulator (EBP family)
*yfi*Q	b2584_1	1	449	pe	NAD(P)-binding module of putative acyl-CoA synthetase
*yfi*Q	b2584_2	464	886	pe	ATP-binding module of putative acyl-CoA synthetase
*phe*A	b2599_1	1	98	e	chorismate mutase P
*phe*A	b2599_2	99	386	e	prephenate dehydratase
*tyr*A	b2600_1	1	100	e	chorismate mutase T
*tyr*A	b2600_2	104	373	e	prephenate dehydrogenase
*bar*A	b2786_1	1	517	e	histidine kinase of hybrid sensory histidine kinase
*bar*A	b2786_2	658	918	r	response regulator of hybrid sensory histidine kinase
*arg*A	b2818_1	1	293	o	acetylglutamate kinase homolog (inactive)
*arg*A	b2818_2	298	442	e	N-alpha-acetylglutamate synthase (amino acid acetyltransferase)
*pts*P	b2829_1	1	159	t	GAF domain containing module of PTS family protein
*pts*P	b2829_2	161	748	t	PTS family enzyme I
aas	b2836_1	1	192	e	2-acylglycerophospho-ethanolamine acyl transferase
aas	b2836_2	198	719	e	acyl-acyl carrier protein synthetase
*ygf*N	b2881_1	1	158	pe	Fe-S binding module of putative selenate reductase
*ygf*N	b2881_2	160	956	pe	molybdopterin-binding module of putative selenate reductase
*ygf*T	b2887_1	1	165	pe	Fe-S binding module of putative oxidoreductase
*ygf*T	b2887_2	173	644	pe	nucleotide-binding module of putative oxidoreductase
*glc*E	b2978_1	1	314	e	FAD binding module of glycolate oxidase
*glc*E	b2978_2	341	761	e	Fe-S binding module of glycolate oxidase
gsp	b2988_1	1	251	e	glutathionylspermidine amidase
gsp	b2988_2	252	619	e	glutathionylspermidine synthetase
*hld*E	b3052_1	1	316	pe	putative kinase module of ADP-L-glycero-D-manno-heptose synthase
*hld*E	b3052_2	326	477	pe	putative sugar nucleotide transferase of ADP-L-glycero-D-manno-heptose synthase
*gln*E	b3053_1	1	423	e	deadenylylase for glutamine synthetase, regulates P-II (GlnB) and GlnK
*gln*E	b3053_2	425	946	e	adenylylase for glutamine synthetase, regulates P-II (GlnB) and GlnK
aer	b3072_1	1	260	r	sensory module of aerotaxis sensor receptor, senses cellular redox state or proton motive force
aer	b3072_2	262	506	r	methyl accepting chemotaxis module of aerotaxis sensor receptor, senses cellular redox state or proton motive force
*inf*B	b3168_1	1	296	pm	putative membrane module of protein chain initiation factor IF-2 module
*inf*B	b3168_2	312	890	f	protein chain initiation factor IF-2

*arc*B	b3210_1	1	502	e	sensory kinase of hybrid sensory histidine kinase
*arc*B	b3210_2	519	776	r	response regulator of hybrid sensory histidine kinase
*yhe*S	b3352_1	1	285	pt	putative transport protein (ABC superfamily, ATP_bind)
*yhe*S	b3352_2	286	637	pt	putative transport protein (ABC superfamily, ATP_bind)
*cys*G	b3368_1	1	213	e	bifunctional: 1,3-dimethyluroporphyriongen III dehydrogenase; siroheme ferrochelatase of siroheme synthase
*cys*G	b3368_2	216	456	e	uroporphyrinogen methyltransferase of siroheme synthase
*mrc*A	b3396_1	1	237	e	transglycosylase of penicillin-binding protein 1a
*mrc*A	b3396_2	267	858	e	transpeptidase of bifunctional penicillin-binding protein 1a
*feo*B	b3409_1	1	166	t	GTP-binding module of ferrous iron transport protein B (FeoB family)
*feo*B	b3409_2	167	773	t	membrane module of ferrous iron transport protein B (FeoB family)
*mal*T	b3418_1	1	673	o	module with possible MalY interaction site
*mal*T	b3418_2	703	901	r	transcriptional activator of maltose utilization (LysR familiy)
*fts*Y	b3464_1	1	185	m	membrane binding module of cell division protein
*fts*Y	b3464_2	186	497	e	GTPase module of cell division protein
*rbb*A	b3486_1	1	235	e	ATP-binding module of ribosome-associated ATPase
*rbb*A	b3486_2	249	517	e	ATP-binding module of ribosome-associated ATPase
*rbb*A	b3486_3	519	893	pm	putative membrane protein of ribosome-associated ATPase
*xyl*G	b3567_1	1	250	t	D-xylose transport protein (ABC superfamily, ATP_bind)
*xyl*G	b3567_2	251	513	t	D-xylose transport protein (ABC superfamily, ATP_bind)
*mtl*A	b3599_1	1	465	t	PTS family enzyme IICB, mannitol-specific
*mtl*A	b3599_2	494	637	t	PTS family enzyme IIA, mannitol-specific
dfp	b3639_1	1	197	e	4'-phosphopantothenoylcysteine decarboxylase
dfp	b3639_2	213	429	e	phosphopantothenoylcysteine synthetase, FMN-binding
*dgo*A	b3692_1	1	202	e	2-dehydro-3-deoxygalactonate 6-phosphate aldolase
*dgo*A	b3692_2	206	587	e	galactonate dehydratase
*bgl*F	b3722_1	1	460	r	PTS family enzyme IIBC, beta-glucoside-specific, cryptic
*bgl*F	b3722_2	467	625	r	PTS family enzyme IIA, beta-glucoside-specific, cryptic
*glm*U	b3730_1	1	244	e	N-acetyl glucosamine-1-phosphate uridyltransferase
*glm*U	b3730_2	269	456	e	glucosamine-1-phosphate acetyl transferase
*yie*N	b3746_1	1	306	pr	putative sigma54 activator protein, enhancer binding protein (EBP family)
*yie*N	b3746_2	307	505	o	unknown function module of putative transcriptional regulator
*rbs*A	b3749_1	1	254	t	high-affinity D-ribose transport protein (ABC superfamily, ATP_bind)
*rbs*A	b3749_2	255	501	t	high-affinity D-ribose transport protein (ABC superfamily, ATP_bind)
*fad*B	b3846_1	1	264	e	multifunctional: 3-hydroxybutyryl-CoA epimerase, delta(3)-cis-delta(2)-trans-enoyl-CoA isomerase, enoyl-CoA hydratase
*fad*B	b3846_2	308	729	e	3-hydroxyacyl-CoA dehydrogenase
*pol*A	b3863_1	1	297	e	5'->3' exonuclease of DNA polymerase I
*pol*A	b3863_2	327	928	e	bifunctional: 3'->5' exonuclease; 3'->5' polymerase of DNA polymerase I
*gln*G	b3868_1	1	120	r	response regulator, two-component regulator with GlnL, nitrogen regulation
*gln*G	b3868_2	139	469	r	sigma54 interaction module of response regulator (EBP family)
*frv*B	b3899_1	1	110	t	PTS family enzyme IIB, fructose-specific
*frv*B	b3899_2	112	485	t	PTS family enzyme IIC, fructose-specific
*met*L	b3940_1	1	454	e	aspartokinase II, methionine sensitive
*met*L	b3940_2	458	810	e	homoserine dehydrogenase II, methionine sensitive
*pts*A	b3947_1	1	558	t	bifunctional PTS family Hpr ; enzyme I
*pts*A	b3947_2	567	711	t	PTS family enzyme IIA
*zra*R	b4004_1	1	125	r	response regulator
*zra*R	b4004_2	140	441	r	sigma54 interaction module of response regulator (EBP family)
*pur*H	b4006_1	1	199	e	IMP cyclohydrolase
*pur*H	b4006_2	221	529	e	phosphoribosylaminoimidazolecarboxamide formyltransferase
*mal*K	b4035_1	1	246	t	maltose transport protein (ABC superfamily, ATP_bind)
*mal*K	b4035_2	247	371	r	regulatory module, repression of mal operon and regulatory interaction with MalT and MalX
*als*A	b4087_1	1	259	t	allose transport protein (ABC superfamily, ATP_bind)
*als*A	b4087_2	260	510	t	allose transport protein (ABC superfamily, ATP_bind)
*dsb*D	b4136_1	1	450	e	thiol:disulfide interchange protein, cytochrome c-type biogenesis, electron carrier

*dsb*D	b4136_2	451	565	e	thiol:disulfide interchange protein, cytochrome c-type biogenesis, electron carrier
*yji*R	b4340_1	1	90	pr	putative regulator
*yji*R	b4340_2	92	470	pe	putative aminotransferase
*yjj*K	b4391_1	1	281	pt	putative transport protein (ABC superfamily, ATP_bind)
*yjj*K	b4391_2	306	555	pt	putative transport protein (ABC superfamily, ATP_bind)

[1]Gene product type: c, carrier; pc, putative carrier; e, enzyme; pe, putative enzyme; f, factor; pf, putative factor; m, membrane protein; pm, putative membrane protein; r, regulatory protein; pr, putative regulatory protein; t, transport protein; pt, putative transport protein; o, unknown function.
